# Apical Revascularization after Delayed Tooth Replantation: An Unusual Case

**DOI:** 10.1155/2016/2651643

**Published:** 2016-11-02

**Authors:** Marília Pacífico Lucisano, Paulo Nelson-Filho, Lea Assed Bezerra Silva, Raquel Assed Bezerra Silva, Fabricio Kitazono de Carvalho, Alexandra Mussolino de Queiroz

**Affiliations:** Department of Pediatric Dentistry, School of Dentistry of Ribeirão Preto, University of São Paulo, Ribeirão Preto, SP, Brazil

## Abstract

The aim of this paper is to present the clinical and radiological outcome of the treatment involving a delayed tooth replantation after an avulsed immature permanent incisor, with a follow-up of 1 year and 6 months. An 8-year-old boy was referred after dental trauma that occurred on the previous day. The permanent maxillary right central incisor (tooth 11) had been avulsed. The tooth was hand-held during endodontic therapy and an intracanal medication application with calcium hydroxide-based paste was performed. An apical plug with mineral trioxide aggregate (MTA) was introduced into the apical portion of the canal. When the avulsed tooth was replanted with digital pressure, a blood clot had formed within the socket, which moved the MTA apical plug about 2 mm inside of the root canal. These procedures developed apical revascularization, which promoted a successful endodontic outcome, evidenced by apical closure, slight increase in root length, and absence of signs of external root resorption, during a follow-up of 1 year and 6 months.

## 1. Introduction

Tooth avulsion is one of the most severe types of trauma, which often affects young permanent dentition [1]. In this type of injury, a tooth is completely displaced from its alveolar socket, affecting the pulp tissue, periodontal ligament, dental hard tissues, and alveolar bone [2]. Although the best therapy for avulsed teeth is immediate replantation [3], it is not always possible in clinical conditions [4]. Consequently, delayed replantation is often required [5].

Replacement [6] and inflammatory external root resorption [7] commonly affect delayed reimplanted teeth. Both are progressive processes that damage the root structure, potentially leading to tooth loss [8].

Recently, revascularization therapy has been proposed as an alternative approach for immature necrotic teeth, with the advantage of inducing root-end development, thickening of radicular dentin, and reinforcement [9, 10].

The revascularization treatment protocol basically involves the following procedures: disinfection of the pulp space with an effective intracanal medication between sessions, commonly using a bi- or triantibiotic paste [11] or a calcium hydroxide-based paste [12, 13]; overinstrumentation to induce bleeding and production of a scaffold into the canal space; and placement of an MTA barrier on the blood clot followed by a tight sealing of the coronal access cavity [11]. It has been proposed that blood clot formation in the canal space is the source of stem cells from the apical papilla [14], which play a key role during wound healing [15].

This study aimed to describe an unusual case in which apical revascularization associated with delayed tooth replantation was performed.

## 2. Case Report

An 8-year-old boy was referred to the Pediatric Dentistry Clinic after dental trauma occurred on the previous day as a result of a bicycle accident. The patient arrived at the dental clinic about 17 hours after the trauma. During the clinical examination, the permanent maxillary right central incisor (tooth 11) had been avulsed, which was kept at an extraoral dry time of 13 hours. After this period, the avulsed tooth was transferred to a bottle with milk. The child was systemically healthy and there was no relevant medical history. A signed, written informed consent form was obtained from the patient's guardian.

Intraoral and radiographic examination revealed that, besides tooth avulsion, extrusion of the permanent maxillary left central incisor (tooth 21), slight intrusion of the permanent maxillary right lateral incisor (tooth 12), and lateral luxation of the permanent maxillary left lateral incisor (tooth 22) had occurred. Additionally, all of these teeth presented uncomplicated crown fractures (involving only enamel).

The chosen treatment sequence for this case is described below.

**Figure 1:**
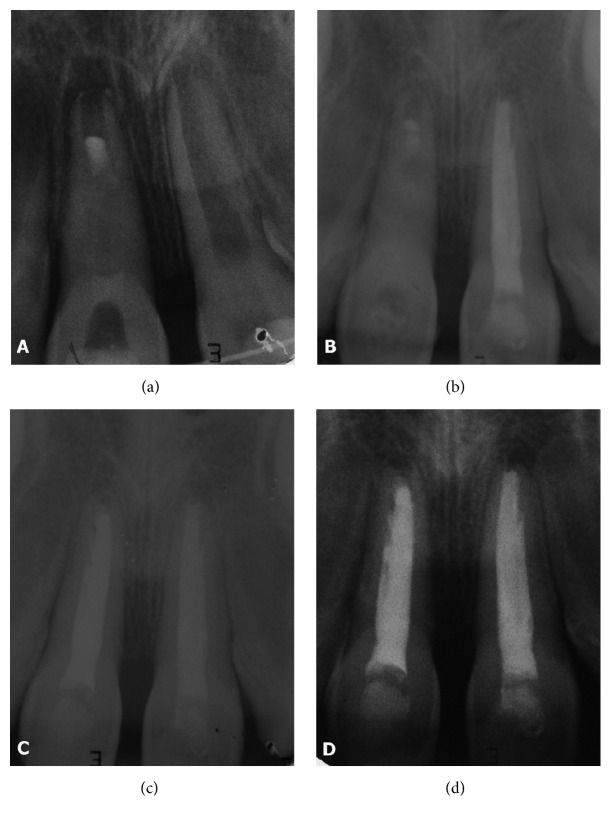
(a) Initial radiograph after tooth replantation (tooth 11), evidencing the incomplete root formation, the MTA apical plug about 2 mm inside of root canal, and the intracanal medication with calcium hydroxide-based paste. (b) Radiographic examination after 2 months, revealing apical closure and slight increase in root length of the replanted tooth (tooth 11). Endodontic treatment was performed on the left central incisor (tooth 21). (c) Radiographic examination 6 months after treatment of tooth 11, revealing apical closure. (d) Final radiographic examination, after a follow-up of 1 year and 6 months, revealing apical closure and no sign of external root resorption of the replanted right central incisor (tooth 11).

First, the avulsed tooth (tooth 11) was gently washed with saline solution and the nonvital periodontal fibers were removed from the root surface. Thereafter, the tooth was hand-held (by the coronal portion) during endodontic therapy, including access cavity preparation, pulpectomy, and root canal instrumentation with a K-file # 80 (Maillefer, Ballaigues, Switzerland) at 1 mm short of the root canal length, under irrigation with 1% sodium hypochlorite (NaOCl). After biomechanical preparation, the root canal was dried with sterile paper points and an intracanal medication with calcium hydroxide-based paste (Calen®, SS White Artigos Dentários Ltda., Rio de Janeiro, RJ, Brazil) was performed. The Calen paste is composed of 2.5 g calcium hydroxide, 0.5 g zinc oxide p.a., 0.05 g colophony, and 2 mL polyethylene glycol 400 (vehicle). Then, the coronal access was temporarily sealed with a sterile cotton pellet and glass-ionomer cement (Vidrion R®, SS White Artigos Dentários Ltda., Rio de Janeiro, RJ, Brazil). After that, mineral trioxide aggregate (ProRoot MTA, Dentsply Tulsa Dental, Tulsa, OK) was introduced into the apical portion of the canal, creating an apical plug, as recommended by the American Academy of Pediatric Dentistry (2014) [16]. No treatment was performed on the root surface after removal of nonviable periodontal ligament fibers [17]. After local anesthesia, the socket was gently curetted to remove any coagulum or granulation tissue and was washed with saline solution. When the avulsed tooth was replanted with digital pressure, a blood clot was formed within the socket, which moved the MTA apical plug about 2 mm to the inside of root canal. This unintentional procedure worked like apical revascularization, since the apical third of the canal was filled with a blood clot rich in stem cells, which were biostimulated by MTA (Figure 1(a)).

The teeth were then prepared for receiving splinting, involving acid etching, application of a bonding agent, and repositioning of the extruded tooth (tooth 21). The teeth were splinted from canine to canine, excluding the slightly intruded right lateral incisor (tooth 12), with composite resin and a 0.4 orthodontic wire. Systemic treatment included 7 days of 250 mg amoxicillin 3 times daily. The patient received instructions to have a soft diet and was informed on the importance of maintaining oral hygiene and the use of chlorhexidine mouthwash was recommended twice a day for 2 weeks. After 4 weeks, the splint was removed and the patient did not present any postoperative clinical or radiographic complications. The enamel fractures were restored with composite resin (Filtek Z250 XT, 3M ESPE, St. Paul, MN, USA).

After one month, it was necessary to endodontically treat the left central incisor (tooth 21) due to negative response to the cold sensitivity test. Endodontic treatment included access cavity preparation, root canal instrumentation up to a K-file # 80 under irrigation with 1% NaOCl, intracanal dressing with calcium hydroxide-based paste for 2 weeks, and obturation with gutta-percha points and a calcium hydroxide-based sealer (Sealapex; Kerr Corporation, Orange, CA, USA).

The patient was followed up regularly and, after 2 months, the radiographic examination revealed that the replanted tooth (tooth 11) showed apical closure and a slight increase in root length (Figure 1(b)).

The replanted tooth (tooth 11) was then obturated with gutta-percha points and a calcium hydroxide-based sealer, up to the limit of the apical plug.

The patient was followed up every month and after 1 year and 6 months. As shown in Figures 1(c) and 1(d), periapical radiographic examination showed apical closure and no sign of external root resorption of the replanted right central incisor (tooth 11). Clinically, the tooth remained with no symptomatology and mobility. Also, the left central incisor (tooth 21) remained with no radiographic signs of external root resorption and the lateral incisors (teeth 12 and 22) have shown continued root development.

## 3. Discussion

Currently, clinical management of delayed tooth replantation still represents a challenge for dentists. The literature has extensively demonstrated that 1 hour of extraoral dry time is critical, and after this period the periodontal ligament (PDL) cells remaining on the root surface of the avulsed tooth are less likely to be viable [7]. According to Guidelines from the International Association of Dental Traumatology, in conditions of dry time longer than 1 hour, like the present case, the PDL cells will be necrotic and are not expected to heal and thus need to be removed. In this situation, the delayed replanted tooth has a poor long-term prognosis and the expected outcome will be replacement due to external root resorption [17].

In the case described in this study, the treatment protocol was based on current guidelines for avulsed permanent teeth, which developed apical revascularization. It is highlighted that when the treatments were performed, pulp revascularization was not yet well established in the literature as a safe and effective treatment. Thus, it was decided to perform conventional treatment for both teeth. The procedure performed on the replanted tooth apparently promoted a successful endodontic outcome, as evidenced radiographically by apical closure and slight increase in root length. This response is consistent with previous reports on pulp revascularization in immature necrotic permanent teeth [12]. Recently, regenerative endodontic procedures have been applied to replanted permanent teeth after a brief [18] or >8 hours' extra-alveolar period [19] with promising results.

It is noteworthy that the revascularization of pulp space and methods to promote it are among the future areas of research on promising treatment procedures for avulsed teeth recommended by Guidelines for the Management of Traumatic Dental Injuries [17].

Revascularization has emerged as a promising alternative treatment to conventional apexification, presenting advantages such as root-end development and radicular reinforcement [10, 20]. The nature of tissues formed in the canal after revascularization therapy was first described by Thibodeau et al. [21] after performing a study in immature dog teeth. It was observed that cementum-, bone-, and periodontal ligament-like tissues were formed, which was subsequently confirmed by other* in vivo* studies [22–24]. According to Simon and Smith [25], the origin of the formed tissue may not be so important from a clinical perspective, since the objective is to induce apical closure and healing of the periapical tissues and to keep the patient free from signs and symptoms, like in this current case report.

Recruitment of stem cells to the injured site and their differentiation into specific tissue-committed cells are required for wound healing [26]. It was suggested that stem cells of apical papilla (SCAP) can be considered an endogenous cell source for pulp revascularization [23, 27]. However, in cases of loss of apical papilla, which probably occurs with avulsed teeth, stem cells can be migrated from the periapical tissues or from a distance source into the canal space [28]. According to Ostby [29], bleeding induced into the root canal space is a source of viable cells, which are derived from circulating cells, cementum, periodontal ligament, or alveolar bone. In the present case, the clinical procedure allowed the blood clot to fill the apical portion of the root canal, which was most likely the source of stem cells for healing and mineralized tissue formation.

Additionally, the procedure performed in the present case enabled direct contact of the blood clot with MTA. As extensively reported in the literature, MTA is a biomaterial with excellent tissue compatibility, good sealing capacity, and a mineralized tissue inducing effect [30, 31], which may have played a key role in the apical closure reached in this case.

It is known that infection control is essential for apical repair of immature teeth [20]. In cases of delayed tooth replantation, endodontic treatment and systemic antibiotic therapy must be performed in order to control the contamination [32], as was carried out in the present case report. The use of an intracanal medication in pulp revascularization is a critical aspect since the disinfection of the root canal should be achieved with minimum mechanical instrumentation [13, 18]. Due to its ideal properties for removal of endodontic infections, induction of a mineralizing effect, and control of root resorptive processes [32], calcium hydroxide-based paste was used as intracanal medication during the root canal treatment performed prior to replantation, as is still currently recommended [13].

Therefore, all efforts and cautions to manage this case were conducted with the aim to improve the prognosis. After 1 year and 6 months, radiographic examination showed apical closure and no sign of external root resorption of the replanted tooth. However, it should be emphasized that in the long term ankylosis and replacement resorption can take place because of the loss of the PDL cells, as is common after performing a delayed replantation [17].

In conclusion, the clinical procedures performed in the present case of delayed replantation of an avulsed immature tooth, which worked like apical revascularization, promoted a successful endodontic outcome after a follow-up of 1 year and 6 months. However, studies with this protocol and a longer period of follow-up are needed to scientifically demonstrate the efficacy and safety of this therapy.
